# Recovery and Purity Trade-Off in Nd/Ho Separation from Real NdFeB Magnet Leachates

**DOI:** 10.3390/ma19183888

**Published:** 2026-09-12

**Authors:** Gorka Barquero-Carmona, Lorena Alcaraz, Olga Rodríguez-Largo, Félix A. López

**Affiliations:** Centro Nacional de Investigaciones Metalúrgicas (CENIM), Consejo Superior de Investigaciones Científicas (CSIC), Avda. Gregorio del Amo, 8, 28040 Madrid, Spain; olga.rodriguez@csic.es (O.R.-L.); f.lopez@csic.es (F.A.L.)

**Keywords:** rare earth elements, selective recovery, hydrometallurgy, NdFeB magnets

## Abstract

The growing demand for electric vehicles has increased the need for rare earth elements (REEs), promoting the recycling of secondary sources such as end-of-life NdFeB permanent magnets. However, the selective separation of REEs with similar chemical properties, such as neodymium (Nd) and holmium (Ho), remains a major challenge. In this work, their separation from a real NdFeB permanent magnet leachate was investigated by liquid–liquid extraction using di-(2-ethylhexyl) phosphoric acid (D2EHPA) as an extractant. The effects of extractant concentration (0.05 M and 0.1 M), organic-to-aqueous phase ratio (1:1 and 2:1, O:A), and pH (0.6 and 2) on the extraction process were evaluated. Increasing extractant concentration and phase ratio improved Ho extraction, reaching above 90%, whereas Nd co-extraction remained relatively low at pH 0.6 (0.8–4.6%, depending on the conditions). However, pH was found to be the key parameter. At pH 2, Ho extraction exceeded 96% in all cases, while Nd co-extraction increased significantly (up to ~28%), reducing selectivity. Due to the high initial Nd:Ho concentration ratio (~12:1), even low Nd co-extraction percentages resulted in significant contamination of Ho-rich products, identifying product purity as the primary limiting factor. These results show a trade-off between recovery and selectivity, with low pH favoring selectivity and high pH favoring extraction efficiency.

## 1. Introduction

The electrification of the transport sector has led to a significant increase in the demand for electric vehicles in recent years, leading to a growing consumption of critical raw materials [1,2]. Among these, rare earth elements (REEs) are essential due to their unique properties [3,4], which are required for high-performance applications [5]. In particular, NdFeB permanent magnets are one of the most important applications of REEs, as they are extensively employed in electric vehicle motors due to their high magnetic strength and efficiency [6].

The extraction of REEs is associated with several problems, such as dependence on other regions and the environmental impact of their extraction and processing [7,8]. In addition, the recovery, separation, and purification of rare earth elements from primary sources require complex metallurgical processes because of their similar chemical behaviors. Therefore, achieving a sustainable and efficient obtention of REEs remains a major challenge.

For this reason, recycling secondary source materials such as end-of-life NdFeB permanent magnets has become a more sustainable alternative [6,9]. These magnets are an excellent starting material because of their high concentration of rare earth elements, mainly neodymium, and other valuable REEs present in smaller quantities. This approach is very promising because it reduces the need for primary mining while lowering environmental impact and dependency on REE supplies.

Despite these advantages, the recovery of REEs from secondary sources remains challenging [10], and their selective separation is even more difficult because of their similarities [11]. However, slight differences in ionic radius caused by the lanthanide contraction enable a differentiation between light (LREEs, e.g., Nd, Pr, Sm) and heavy rare earth elements (HREEs, e.g., Ho, Dy, Gd, Tb, Er), allowing their selective separation [12].

Based on these slight differences, several hydrometallurgical methods have been proposed for the selective separation of REEs, including precipitation, ion exchange, adsorption, and membrane-based processes [13,14]. However, solvent extraction remains the most widely used method due to its high efficiency and scalability at the industrial level [15].

Among the different extractants studied, organophosphorus-based compounds are the most commonly used for REE separation [11,16]. These include acidic extractants such as di-(2-ethylhexyl) phosphoric acid (D2EHPA), phosphonic acids (e.g., PC 88A), and phosphinic acids (e.g., Cyanex 272), which have demonstrated high extraction efficiency and selectivity under controlled conditions [17,18]. The extraction performance strongly depends on key operational parameters, such as the amount of extractant and aqueous phase pH, which directly influence extraction equilibrium and selectivity.

Nevertheless, most studies reported in the literature are carried out using synthetic solutions, which do not fully represent the complexity of real systems (e.g., residual Fe and B) or variability in REE ratios present in real leachates [19,20,21]. These factors can alter the extraction equilibria and selectivity, making it difficult to translate the findings from synthetic systems to industrial conditions.

This study addresses the selective separation of neodymium and holmium from a real leachate obtained from end-of-life NdFeB magnets in electric-vehicle motors. Alcaraz et al. [22] demonstrated promising selectivity at 0.05 M D2EHPA, an organic-to-aqueous phase ratio (O:A) of 1:1, and pH 0.6, but did not systematically investigate the effects of these parameters or quantify trade-offs between recovery and purity.

To evaluate the effect of these parameters, we conducted a systematic investigation of liquid–liquid extraction using D2EHPA, varying extractant concentration (0.05 M and 0.1 M as monomer), organic-to-aqueous phase ratio (1:1 and 2:1), and pH (0.6 and 2), and characterized the resulting REE oxalates to assess both extraction efficiency and product purity.

## 2. Materials and Methods

### 2.1. Preparation of REE Solution

As seen in a previous study [22], the recovery of REEs from NdFeB permanent magnets begins with the previously demagnetized magnet, which was then milled by hydrogen decrepitation. This magnet powder underwent chlorination roasting at 300 °C for 3 h. Subsequently, the resulting solid was leached in water at 80 °C for 1 h, obtaining a suspension that was subsequently filtered. The recovery of rare earth elements from the initial magnet was higher than 90%. The obtained solution underwent liquid–liquid extraction and was treated with Aliquat 336 diluted in Solvesso to extract the iron present in the solution. The resulting solution was enriched in REEs and then used in the selective recovery experiments.

### 2.2. REE Selective Recovery Experiments

The separation of the REEs (Nd/Ho) was carried out by liquid–liquid extraction using D2EHPA as the Ho extractant diluted in Exxsol D100. D2EHPA was selected because it has been shown to preferentially extract HREEs over LREEs under acidic conditions [23] and was used in the prior work by Alcaraz et al. [22], allowing a direct comparison of the investigated operating conditions. All extraction experiments were performed at 25 °C with a contact time of 30 min while varying key parameters, including extractant concentration, organic-to-aqueous phase ratio, and aqueous phase pH. The contact time was selected at 30 min based on preliminary kinetic experiments performed at different extraction and stripping times. In all cases, extraction efficiencies reached a plateau after approximately 30 min, indicating that equilibrium conditions had been effectively achieved.

All extraction and stripping experiments were carried out in 1 L jacketed glass separatory funnels equipped with a mechanical stirrer operating at 250 rpm.

The pH was adjusted (initial pH 0.6 to pH 2) by small additions of 30% NH_3_ and was continuously monitored with a pH meter. Initial and equilibrium aqueous-phase pH values were measured, and the obtained values before and after extraction are now included. Thus, a system with an initial pH of 2.0 dropped to pH 1.7 after extraction, while at an initial pH of 0.60, the change was insignificant (final pH 0.59).

Finally, the Nd-rich aqueous phase was treated with oxalic acid to precipitate neodymium oxalate. Simultaneously, the organic phase was treated with a saturated oxalic acid solution at an organic-to-aqueous phase ratio of 1:1, at 25 °C for 30 min to strip Ho as oxalate. During this stripping–precipitation stage, no significant phase-separation difficulties or persistent emulsions were observed. After contact, the organic and aqueous phases separated readily, and the precipitated rare earth oxalate was easily recovered from the aqueous phase by filtration. The stripping–precipitation step resulted in a recovery of more than 99% of the extracted REEs under all investigated conditions.

### 2.3. Characterization

Rare earth content in the solutions was analyzed by Inductively Coupled Plasma–Optical Emission Spectrometry (ICP-OES) using an Agilent ICP-OES (Agilent Technologies, Santa Clara, CA, USA), model 5100 VDV (Vertical Dual View).

The composition of the solids was determined through microwave-assisted digestion using a Speedwave Xpert Microwave System (Berghof Products + Instruments GmbH, Eningen unter Achalm, Germany). For this, 0.1 g of each sample was put in contact with a mixture of HNO_3_:HCl (1:3) and subjected to heating until reaching 240 °C under a pressure of 80 bar. The liquid obtained for each test was transferred to a 100 mL flask and brought to volume using Milli-Q water (Merck Millipore, Burlington, MA, USA).

pH measurements were performed using a Sension+ pH3 pH meter (Hach, Loveland, CO, USA) equipped with a 5010T pH electrode.

Structural characterization was carried out via X-ray diffraction (XRD) using an X’Pert Pro MPD diffractometer (PANalytical, Almelo, The Netherlands) equipped with a Cu anode (Cu Kα radiation) with a step size of 0.03° (2θ) in the range 10°–70°.

The shape of the samples was examined using scanning electron microscopy (SEM) with a JEOL JSM-IT700HR microscope (JEOL Ltd., Tokyo, Japan), along with an energy-dispersive X-ray microanalyzer (EDX). For SEM viewing, the powdered samples were placed on a sticky conductive carbon disk.

## 3. Results and Discussion

The metal content in the solution (mg/L) after the leaching and iron removal steps is shown in Table 1.

The results show the successful removal of Fe and the purity of the REE solution. The predominant REEs are neodymium and holmium. However, the concentration of neodymium is approximately 12 times higher than that of holmium, making their separation even more challenging. This significant difference in the initial composition implies that even small fractions of Nd co-extraction can result in considerable contamination of the final Ho product, making purity the most critical parameter in this process. This solution was used in the separation experiments.

A systematic study of Nd/Ho separation was carried out by varying three key parameters: (i) extractant concentration (0.05 M and 0.1 M), (ii) organic-to-aqueous phase ratio (1:1 and 2:1, O:A), and (iii) pH (0.6 and 2).

### 3.1. Effect of D2EHPA Concentration

The initial study examined two D2EHPA concentrations (0.05 M and 0.1 M) under constant conditions: an O:A ratio of 1:1 and the original aqueous pH (~ 0.6). The REE extraction percentages are presented in Table 2.

Doubling the D2EHPA concentration from 0.05 M to 0.1 M results in a significant increase in HREE extraction efficiency (from ~54% to ~92% in the case of Ho), consistent with the cation-exchange mechanism in REE extraction. Based on the established literature, extraction is believed to occur through coordination between REE cations and D2EHPA present in its dimeric form, (HA)_2_, as shown in Equation (1) [16,24].

(1) $${REE}_{\left(aq\right)}^{3+}+{3({HA)}_{2}}_{\left(org\right)}⇆{REE({HA_{2})}_{3}}_{\left(org\right)}+{3H^{+}}_{\left(aq\right)}$$

The increased availability of the extractant in the organic phase shifted the equilibrium toward REE–complex formation. However, this enhancement was accompanied by a slight loss of selectivity, as increasing the D2EHPA concentration to 0.1 M resulted in a low co-extraction of neodymium (~1.20%), despite its preference for heavier REEs. This result shows that a higher extractant concentration facilitates the extraction of light REEs into the organic phase.

At 0.05 M D2EHPA and a 1:1 O:A ratio, Ho extraction remained moderate (~54%), while Nd co-extraction was negligible (<1%), establishing a baseline condition of high selectivity but incomplete recovery.

### 3.2. Effect of Organic-to-Aqueous Phase Ratio

Having established that higher extractant concentrations improve Ho extraction but increase Nd co-extraction, we next investigated whether adjusting the organic-to-aqueous phase ratio could enhance recovery while maintaining selectivity. To this end, the influence of the phase ratio was studied by increasing the organic-to-aqueous ratio from 1:1 to 2:1 at a constant pH of 0.6, evaluating both extractant concentrations (Table 3).

At 0.05 M, increasing the phase ratio substantially improved the HREE extraction, from 54.0% (1:1) to 81.4% (2:1) for Ho. This enhancement can be directly attributed to the higher amount of D2EHPA available in the system, which doubled the moles of extractant, thus favoring the transfer of REEs to the organic phase.

A similar trend was observed at 0.1 M, where holmium extraction increased from 91.9% (1:1) to 96.3% (2:1), although the improvement was less pronounced because the extraction efficiency was already high at this concentration.

Interestingly, the system with 0.05 M at a 2:1 ratio can be directly compared with 0.1 M at a 1:1 ratio because both conditions have the same amount of D2EHPA. Despite this, the holmium extraction was higher in the 0.1 M (1:1) system (91.9%) than in the 0.05 M (2:1) system (81.4%). This suggests that the extraction efficiency may be influenced not only by the total amount of extractant but also by its concentration in the organic phase, which in turn affects complexation equilibria and mass transfer. Furthermore, neodymium co-extraction remains low in both cases (~ 1.20%), indicating that increasing phase availability does not significantly promote its co-extraction.

### 3.3. Effect of pH

The effect of pH was evaluated by repeating all four previous experiments at pH 2, which was found to have the strongest impact on the extraction behavior of the samples (Table 4). While extractant concentration and phase ratio improved Ho recovery, both parameters showed limited ability to enhance selectivity. We therefore investigated pH as a potentially dominant parameter, hypothesizing that it would exert stronger control over the extraction equilibrium and Nd/Ho discrimination.

At pH 2, holmium extraction exceeded 96% in all cases, regardless of extractant concentration or phase ratio. There was also a significant increase for the rest of the HREEs. This confirms that the increase in pH is the dominant factor controlling extraction efficiency.

In contrast, neodymium co-extraction is strongly dependent on both pH and system conditions. Increasing pH from 0.6 to 2 led to a noticeable increase in Nd extraction, which further increased with both extractant concentration and phase ratio. At pH 2, Nd extraction ranged from 3.70% (0.05 M, 1:1) to 28.8% (0.1 M, 2:1).

Additionally, analogous conditions with the same D2EHPA amount (0.05 M at 2:1 and 0.1 M at 1:1) show very similar behavior at pH 2. Holmium extraction exceeded 96% in both cases, whereas neodymium extraction remained comparable (7.52% and 6.00%, respectively). This further confirms that at higher pH, the system may be governed by the amount of extractant rather than by its distribution between phases.

The observed behavior is consistent with the cation-exchange mechanism described in Equation (1), where a lower proton concentration shifts the equilibrium toward the formation of the REE–D2EHPA complex and the release of protons.

These results highlight that although higher pH improves the overall extraction efficiency, it also reduces selectivity between Ho and Nd (reducing LREE vs. HREE selectivity). Therefore, lower pH conditions (pH 0.6) were more favorable for selective separation, whereas higher pH conditions maximized total extraction.

To further quantify extraction performance and selectivity, distribution ratios (D) and separation factors (β_Ho/Nd_) were calculated according to Equations (2) and (3), respectively.

(2) $$D=\frac{E}{100-E}\cdot \frac{A}{O}$$

(3) $$\beta_{Ho/Nd}=\frac{D_{Ho}}{D_{Nd}}$$

where E is the extraction efficiency (%), and A/O is the aqueous-to-organic phase ratio. Accordingly, A/O equals 1 for O:A = 1:1 and 0.5 for O:A = 2:1.

The calculated distribution ratios and separation factors are summarized in Table 5.

In general, D values greater than 1 indicate preferential transfer to the organic phase, whereas values below 1 indicate that the metal remains predominantly in the aqueous phase.

The calculated D values confirm the preferential extraction of Ho over Nd. Ho exhibited D values ranging from 1.17 to 110.1. While the lowest value corresponds to only a moderate transfer of Ho to the organic phase, D values above 10, obtained under most operating conditions, indicate highly favorable extraction. In contrast, Nd showed very low distribution ratios (0.006–0.202), confirming that most of the neodymium remained in the aqueous phase.

The separation factor (β_Ho/Nd_) provides a quantitative measure of the Ho/Nd selectivity of the extraction process. Higher β values indicate greater selectivity towards Ho.

The calculated β_Ho/Nd_ values ranged from 145.6 to 1399.1, confirming the strong preference of D2EHPA for Ho extraction over Nd extraction.

However, despite the high separation factors obtained, significant Nd contamination was still observed in some Ho-rich products. This behavior is mainly related to the feed composition, since Nd was present in a much larger concentration than Ho. Consequently, even small percentages of Nd co-extraction may substantially affect the composition of the Ho-rich product. These results indicate that high separation factors alone are not sufficient to guarantee high product purity when the major component is present in large excess.

These results show the importance of evaluating both extraction selectivity and final product composition. Therefore, characterization of the recovered oxalates is necessary to confirm the quality of the recovered products.

### 3.4. Characterization of REE Oxalates

The extraction efficiency data presented in Section 3.1, Section 3.2 and Section 3.3 provide insight into the partitioning of REEs between phases, but the true measure of separation performance is the purity of the recovered products. To evaluate this, we precipitated REE oxalates from both the Nd-rich aqueous phase and the Ho-rich, loaded organic phase and characterized their composition and structure. This analysis reveals the practical implications of the observed extraction selectivities, particularly the impact of the high initial Nd:Ho ratio on final product purity.

The REE composition (wt.%) of the Nd and Ho oxalates is shown in Table 6 and Table 7, respectively.

The Nd oxalate compositions remained relatively stable across most experimental conditions, showing high Nd content (approximately 38–43 wt.%) and minimal Ho contamination (generally less than 1 wt.%). In contrast, the composition of Ho oxalate is highly sensitive to extraction conditions, particularly Nd co-extraction. At a low pH (~ 0.6), the Nd content in Ho oxalate remains low (about 1–3 wt.%), suggesting that holmium extraction is selective and that the final product maintains relatively high purity.

However, as pH increased, the selectivity decreased significantly, as reflected in the composition of the precipitated Ho oxalate. Even with a low neodymium co-extraction (approximately 3%), the Nd content in Ho oxalate increased markedly to approximately 15 wt.%. Under more extreme conditions, with a neodymium extraction of 28%, the Ho oxalate composition reached approximately 29 wt.% Nd, in some cases, surpassing the Ho content.

Although the results confirm the good extraction performance and selectivity of the process, they do not fully explain the purity of the final products. Due to the high initial excess of neodymium, even low Nd extraction percentages resulted in significant contamination of the Ho oxalate.

A similar trend was observed for the remaining REEs. In general, LREEs followed the behavior of Nd, whereas HREEs followed that of Ho. However, the impact of LREEs (Pr and Sm) on the purity of the Nd oxalate was negligible due to their very low concentrations in the initial solution with respect to the Nd concentration.

In contrast, the higher concentrations of HREEs in the feed solution contributed noticeably to the composition of the Ho-rich product. In addition to Ho, the final oxalate contained appreciable amounts of Tb and Dy, reaching approximately 2 wt.% and 1 wt.%, respectively, under the selected conditions. This indicates that the separation process is particularly effective for concentrating HREEs into the same product while discriminating among the LREEs.

Boron was also monitored throughout the recovery process. No boron extraction or co-precipitation was observed under any of the investigated conditions, indicating that boron did not interfere with the recovery of rare earth elements.

**Figure 1:**
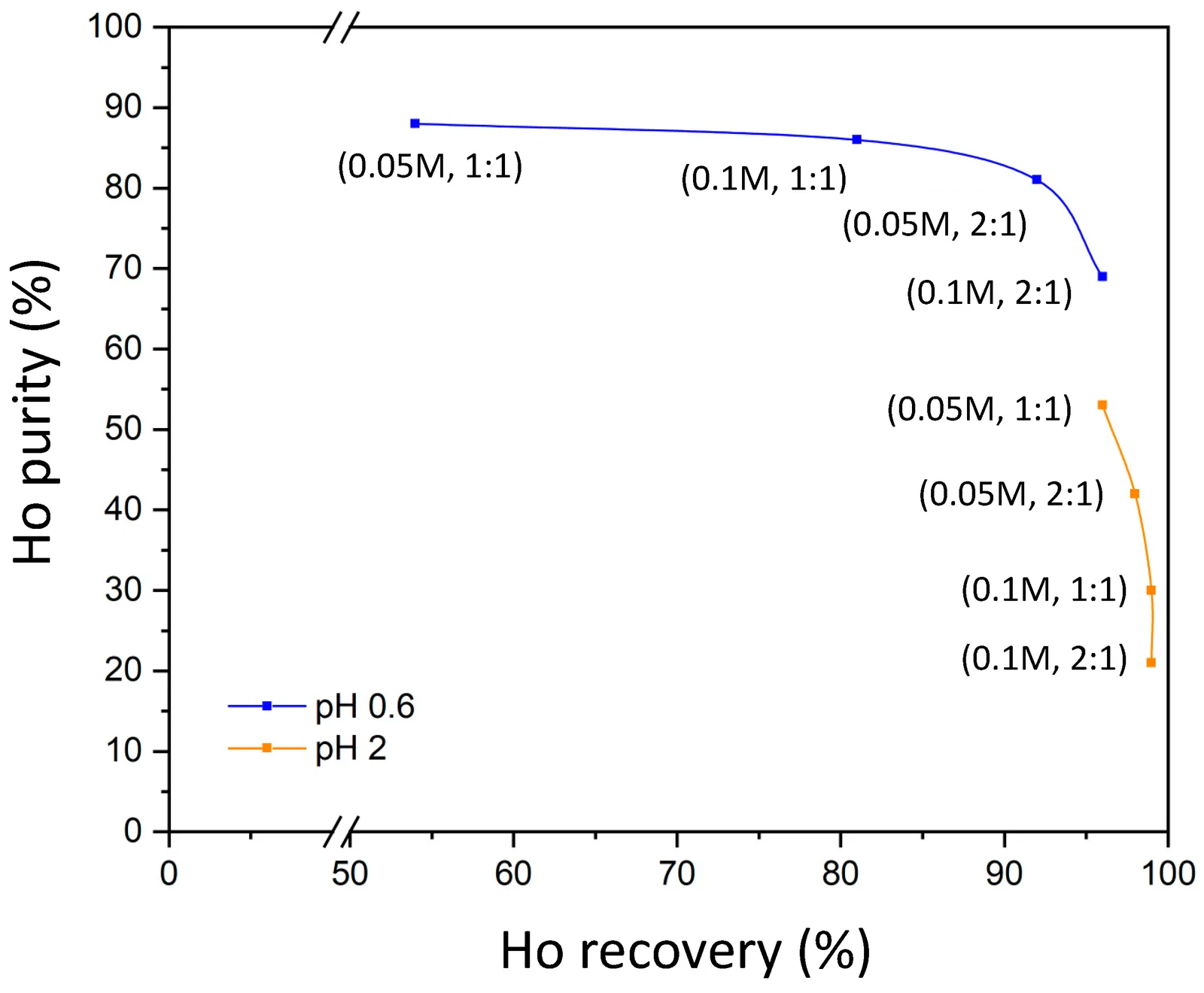
Relationship between Ho purity and Ho recovery for all investigated extraction conditions.

To facilitate visualization of the relationship between extraction performance and product quality, Figure 1 presents the relationship between Ho purity and Ho recovery for all investigated operating conditions. The purity of the Ho-rich product was calculated as the percentage of Ho relative to the total rare earth content present in the corresponding oxalate.

The figure clearly shows the recovery-purity trade-off observed in this study. Under pH 0.6 conditions, substantial improvements in Ho recovery were achieved with only moderate losses in product purity. In contrast, under pH 2 conditions, slight increases in Ho recovery were accompanied by a dramatic decrease in Ho purity due to increased Nd co-extraction. This behavior confirms that product purity becomes the critical limiting factor when Nd is present at much higher concentrations than Ho in the feed solution.

To further characterize the recovered products, the highest-purity Nd-rich and Ho-rich oxalates obtained during the study were selected for X-ray diffraction (XRD) and scanning electron microscopy (SEM) analyses. These products were chosen to confirm the crystal structure, composition, and morphology of the separated rare earth compounds under the most selective operating conditions. The Nd-rich oxalate was obtained under the conditions that maximized Nd purity (0.1 M D2EHPA, O:A = 1:1 and pH 2), whereas the Ho-rich oxalate was obtained under the conditions that maximized Ho purity (0.05 M D2EHPA, O:A = 1:1 and pH 0.6).

**Figure 2:**
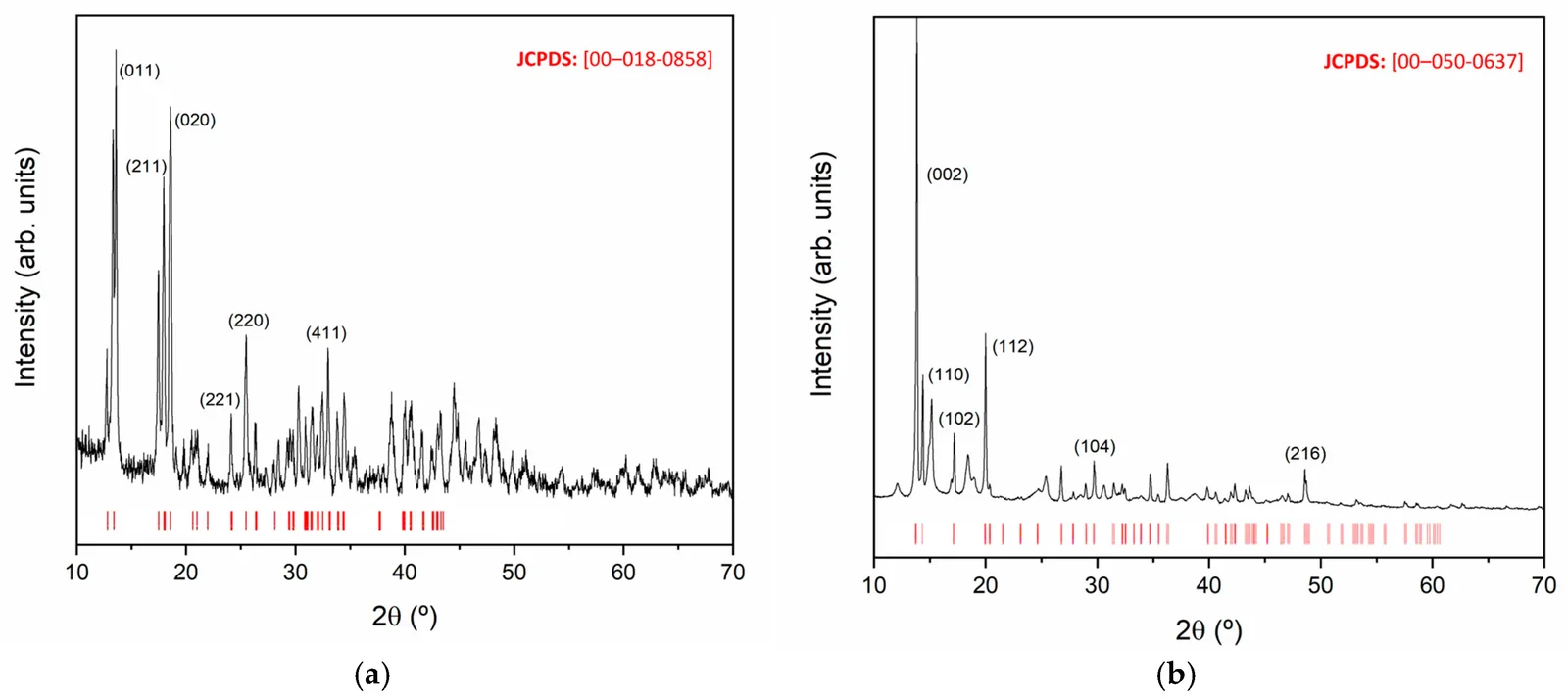
XRD patterns of the obtained (**a**) Nd and (**b**) Ho oxalates.

Figure 2 shows the XRD patterns of the neodymium and holmium oxalate samples. The XRD pattern of the neodymium oxalate (Figure 2a) exhibits diffraction peaks corresponding to Nd_2_(C_2_O_4_)_3_·10H_2_O [JCPDS 00–018-0858]. Similarly, the holmium oxalate (Figure 2b) presents diffraction maxima that can be indexed to Ho_2_(C_2_O_4_)_3_·3H_2_O [JCPDS 00–050-0637]. No additional phases were detected in either sample within the sensitivity of the technique.

**Figure 3:**
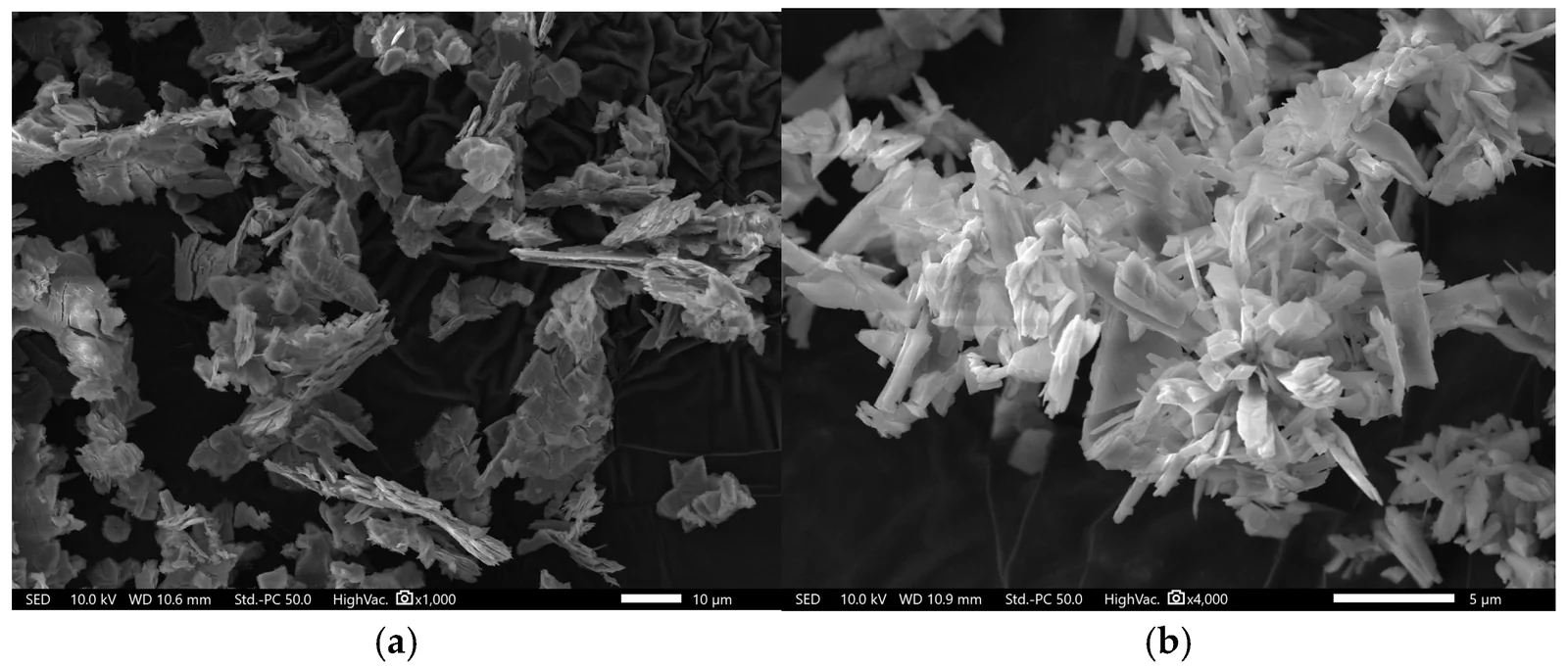
SEM images of the obtained (**a**) Nd and (**b**) Ho oxalates.

Figure 3 shows representative SEM images of the neodymium and holmium oxalates (a and b, respectively). Neodymium oxalate presented thin, plate-like particles arranged in layered structures and irregular orientations, forming agglomerates approximately 10–30 μm in size. In contrast, the Ho oxalate sample displayed more regular polygonal particles, forming agglomerates with sizes in the range of 10 μm. These morphologies are consistent with those previously reported for rare earth oxalates obtained by oxalic acid precipitation [22].

**Figure 4:**
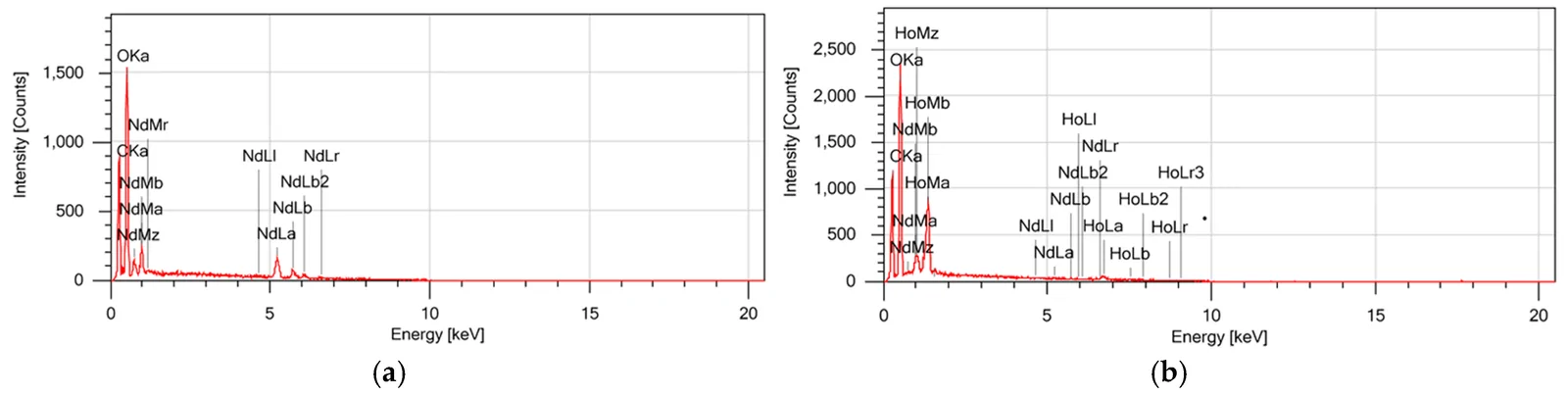
EDX spectra of the obtained (**a**) Nd and (**b**) Ho oxalates.

Figure 4 shows the corresponding EDX spectra of the neodymium and holmium oxalates (a and b, respectively). Neodymium oxalate exhibited only Nd peaks, with no detectable impurities or other REEs. In contrast, Ho was the main element detected in the holmium oxalate spectrum, although small Nd peaks were also detected. This observation is consistent with the ICP-OES results, which indicated a limited amount of Nd in the Ho-rich oxalate. EDX analyses conducted over several representative areas of the samples provided similar elemental compositions, indicating a relatively homogeneous distribution of the major rare earth elements within the recovered oxalates.

## 4. Conclusions

This study demonstrated the feasibility of selectively separating neodymium and holmium from a real NdFeB magnet leachate using D2EHPA extractant. The results confirmed that the extraction performance was strongly influenced by the extractant concentration, phase ratio, and pH.

Increasing D2EHPA concentration and phase ratio improved Ho extraction due to the higher extractant availability, although differences between equivalent extractant amounts revealed that concentration in the organic phase plays a key role. Among all parameters, pH was identified as the dominant factor, enabling nearly complete Ho extraction (>96%) under all conditions at pH 2.

However, a higher pH significantly increased Nd co-extraction, thereby reducing selectivity. This resulted in a trade-off between recovery and selectivity, with low pH favoring selective separation and high pH maximizing the extraction efficiency.

A key challenge in separating REEs from real leachates is that the high concentration of major components (e.g., Nd) relative to minor components (e.g., Ho) means that even low co-extraction percentages can result in severe product contamination. This study quantifies this effect and demonstrates that product purity, rather than extraction efficiency, is the primary limiting factor in such systems.

## Figures and Tables

**Table 1:** Chemical composition of the initial REE solution.

Element	Concentration (mg/L)
Fe	4.40
B	170
Nd	6280
Ho	500
Dy	72.1
Er	28.6
Gd	61.9
Tb	140
Pr	11.2
Sm	17.0

**Table 2:** Extraction efficiency (%) at 1:1 organic-to-aqueous phase ratio at 0.05 M and 0.1 M extractant concentrations (pH 0.6).

	Extraction (%)	
	0.05 M	0.1 M
Nd	0.80	1.20
Ho	54.0	91.9
Dy	29.4	69.1
Er	1.00	27.2
Gd	~0	63.5
Tb	14.3	72.3
Pr	~0	~0
Sm	2.20	~0

**Table 3:** Extraction efficiency (%) at 2:1 organic-to-aqueous phase ratio at 0.05 M and 0.1 M extractant concentrations (pH 0.6).

	Extraction (%)	
	0.05 M	0.1 M
Nd	1.20	4.61
Ho	81.4	96.3
Dy	62.9	~100
Er	11.7	23.7
Gd	56.7	~100
Tb	53.5	84.7
Pr	~0	~0
Sm	~0	4.60

**Table 4:** Extraction efficiency (%) at 1:1 and 2:1 organic-to-aqueous phase ratios at 0.05 M and 0.1 M extractant concentrations (pH 2).

	Extraction (%)			
	0.05 M (1:1)	0.05 M (2:1)	0.1 M (1:1)	0.1 M (2:1)
Nd	3.70	7.50	7.30	28.8
Ho	96.1	98.1	99.1	99.4
Dy	82.3	82.9	83.1	~100
Er	21.7	24.6	30.1	33.4
Gd	67.3	64.7	~100	~100
Tb	90.5	96.9	97.5	~100
Pr	~0	~0	~0	~0
Sm	~0	~0	~0	32.5

**Table 5:** Distribution ratios (D) and separation factors (β_Ho/Nd_) for the investigated extraction conditions.

	pH 0.6				pH 2			
	0.05 M (1:1)	0.05 M (2:1)	0.1 M (1:1)	0.1 M (2:1)	0.05 M (1:1)	0.05 M (2:1)	0.1 M (1:1)	0.1 M (2:1)
DHo	1.2	2.2	11.3	13.0	24.6	25.8	110.1	82.8
DNd	<0.1	<0.1	<0.1	<0.1	<0.1	<0.1	<0.1	0.2
β_Ho/Nd_	145.6	359.0	935.6	537.7	641.7	637.4	1399.1	409.7

**Table 6:** REE composition (wt.%) of the Nd and Ho oxalates obtained from extraction experiments performed at pH 0.6.

	0.05 M (1:1)		0.05 M (2:1)		0.1 M (1:1)		0.1 M (2:1)	
Oxalates	Nd	Ho	Nd	Ho	Nd	Ho	Nd	Ho
Nd	38.5	1.40	41.6	1.78	41.0	3.04	37.7	9.0
Ho	1.20	32.2	0.77	34.2	0.34	34.9	0.20	34.3
Dy	0.14	1.05	0.22	1.50	0.15	1.43	0.05	1.78
Er	0.08	0.56	0.17	0.20	0.16	0.31	0.08	0.53
Gd	0.12	0.18	<L.D.	0.20	<L.D.	1.35	0.08	1.64
Tb	0.33	1.07	0.54	2.01	0.31	2.12	0.07	2.20
Pr	<L.D.	<L.D.	<L.D.	<L.D.	<L.D.	<L.D.	<L.D.	<L.D.
Sm	<L.D.	<L.D.	<L.D.	<L.D.	<L.D.	<L.D.	<L.D.	<L.D.

L.D.: limit of detection.

**Table 7:** REE composition (wt.%) of the Nd and Ho oxalates obtained from extraction experiments performed at pH 2.

	0.05 M (1:1)		0.05 M (2:1)		0.1 M (1:1)		0.1 M (2:1)	
Oxalates	Nd	Ho	Nd	Ho	Nd	Ho	Nd	Ho
Nd	42.8	15.02	43.1	18.18	41.2	21.27	39.90	29.27
Ho	0.20	21.11	0.10	16.18	0.02	11.17	0.04	9.13
Dy	0.32	1.29	0.09	1.84	0.05	1.26	0.09	1.96
Er	0.18	0.32	0.17	0.18	0.08	0.28	0.16	0.36
Gd	<L.D.	0.1	<L.D.	0.72	0.08	0.81	0.17	1.16
Tb	0.60	2.09	0.04	2.03	0.02	2.12	0.02	2.30
Pr	<L.D.	<L.D.	<L.D.	<L.D.	<L.D.	<L.D.	<L.D.	<L.D.
Sm	<L.D.	<L.D.	<L.D.	<L.D.	<L.D.	<L.D.	<L.D.	<L.D.

L.D.: limit of detection.

## Data Availability

The original contributions presented in this study are included in the article. Further inquiries can be directed to the corresponding authors.
